# Dual Viral Infections and Para-Pneumonic Effusion in a Pediatric Patient: A Case of Respiratory Syncytial Virus and COVID-19 Complication

**DOI:** 10.7759/cureus.76176

**Published:** 2024-12-22

**Authors:** Abdullah M Alshushan, Amal Yousif, Saad Almodameg, Khalid Zaydan, Bilal Sayed, Mohammed A Ibrahem, Mohamad-Hani Temsah

**Affiliations:** 1 Alqassim Health Cluster, Ministry of Health in Saudi Arabia, Alqassim, SAU; 2 Emergency Department/Pediatric Emergency Department, King Saud University Medical City, Riyadh, SAU; 3 Pediatric Emergency Medicine Department, King Khalid University Hospital, Alriyadh, SAU; 4 Pediatric Intensive Care Unit, King Khalid University Hospital, Alriyadh, SAU; 5 Pediatric Intensive Care Unit, Pediatric Department, King Saud University Medical City, College of Medicine, King Saud University, Riyadh, SAU

**Keywords:** complicated pneumonia, covid-19, dual viral infection, para-pneumonic effusion, pediatric intensive care unit (picu), pediatric pneumonia, respiratory syncytial virus

## Abstract

Para-pneumonic effusion in children is often associated with bacterial infections; however, dual viral infections, including respiratory syncytial virus (RSV) and COVID-19, can also lead to severe respiratory complications, as demonstrated in this case. This case report presents the clinical course of a pediatric patient with both RSV and COVID-19 infections, leading to para-pneumonic effusion.

A three-year-old girl with a history of asthma and prior febrile convulsions presented to the Emergency Department with fever, cough, vomiting, and fatigue. Chest X-ray revealed left-sided pleural effusion. Initial laboratory results showed elevated inflammatory markers, including a white blood cell count of 22 x 10^9^/L, C-reactive protein (CRP) of 319 mg/L, and procalcitonin of 3.9 ng/mL. Nasal swab polymerase chain reaction confirmed RSV and COVID-19 co-infection. The patient was treated with intravenous ceftriaxone, azithromycin (to cover atypical pathogens), vancomycin (to address possible MRSA [methicillin-resistant *Staphylococcus aureus*]), and corticosteroids to manage severe inflammation with COVID-19. Following a six-day stay in the Pediatric Intensive Care Unit (PICU) for respiratory support and intravenous therapy, the patient showed significant clinical improvement. Serial imaging demonstrated a reduction in pleural effusion, and inflammatory markers decreased markedly. The patient was discharged after 29 days of hospitalization on oral antibiotics, in stable condition, with a follow-up scheduled.

This case underscores the potential severity of dual viral infections in pediatric patients and the importance of prompt diagnosis and comprehensive management to prevent complications such as para-pneumonic effusion.

## Introduction

Para-pneumonic effusion in children is most often associated with bacterial lung infections, with common bacterial pathogens including *Streptococcus pneumoniae*, *Staphylococcus aureus*, and *Streptococcus pyogenes*. Although, primarily, bacterial and viral causes such as infections by respiratory syncytial virus (RSV) and influenza can rarely lead to para-pneumonic effusion. These viral infections can cause inflammation in the lungs, which may predispose to secondary bacterial infections, ultimately contributing to effusion development [1]. The widespread use of vaccines, particularly the pneumococcal conjugate vaccine, has reduced the incidence of bacterial-related cases, but the role of viral agents, particularly during peak winter respiratory seasons, continues to be a consideration [2]. Epidemiologically, para-pneumonic effusion tends to occur more frequently in younger children and coincides with higher rates of respiratory infections in the colder months [3].

Para-pneumonic effusions occur when inflammation from a lung infection extends across the visceral pleura into the pleural cavity, triggering fluid exudation rich in neutrophils and proteins. The progression of an effusion is characterized by increased vascular permeability due to inflammation, which raises the risk of bacterial superinfection, potentially leading to complicated effusion or empyema. Additionally, COVID-19 may exacerbate these respiratory conditions through altered immune responses and interactions with other pathogens, providing valuable insights for clinicians and researchers [4]. This process is driven by increased capillary permeability influenced by cytokines such as interleukin-8 (IL-8) and tumor necrosis factor-alpha (TNF-α). The progression from simple effusion to empyema follows a continuum of three stages: exudative, fibrinopurulent, and organizing stages. The transition from exudative to the fibrinopurulent phase involves an imbalanced cytokine activity and increased leukocytes within the pleural fluid, leading to fibrin accumulation. As inflammation and infection persist, fibrin clots and membranes form within 10 days, resulting in loculated fluid collections. Without timely and effective intervention, fibroblasts can transform the fibrin deposits into a dense, inelastic pleural layer, causing a condition known as trapped lung, which impairs respiratory function [5]. In some cases, bacterial invasion can lead to empyema, characterized by the presence of pneumatoceles, pus formation, and pleural thickening or septations.

Despite advancements in vaccination and medical treatment, para-pneumonic effusion remains a notable cause of morbidity in pediatric populations, underscoring the importance of early detection and management. 

## Case presentation

The patient is a three-year-old girl, 18.5 kg, with a known medical history of seasonal asthma and previous allergies to egg and penicillin, manifesting as urticarial rashes. She has a family history of asthma and did not require previous hospitalization. She had a single episode of febrile convulsions in infancy, with no subsequent seizures or need for antiepileptic medications. She presented to our Emergency Room (ER) with a one-week history of fever, vomiting, cough, and increasing fatigue. Her parents were alarmed by her persistent symptoms, especially since she looked tired and had been visiting different hospitals over the past week associated with mild fever. However, over the day, the child started to have a persistent fever reaching 39°C, responding to anti-pyretic associated with post-tussive vomiting, upper abdominal pain, and poor oral intake. History included recent contact with sick relatives with upper respiratory tract infection (sister) last week with a strong family history of asthma and eczema and immunization history (as per the paper provided, most vaccines were received, but missing vaccines of Rota, +1 dose of pneumococcal, and +1 dose of measles). 

**Figure 1:**
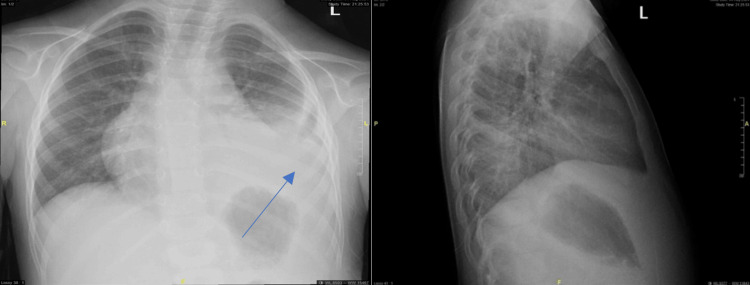
Chest X-ray upon presentation to the Emergency Room (AP, Lat) demonstrating hemogenic opacity on the left lower zone AP, anteroposterior; Lat, lateral.

Her vital signs in the ER showed a temperature of 37.7°C, a heart rate of 141 beats/minute, oxygen saturation of 96% on room air, and a respiratory rate of 44 breaths/minute. She appeared tired with warm extremities and a capillary refill time of less than 2 seconds. On examination, she was leaning on her left side, with decreased air entry on the left side of her chest. An X-ray revealed pleural effusion on the left side, which warranted further investigation and management (Figure 1). The laboratory results showed an elevated white blood cell count of 22 x 10^9^/L, a hemoglobin level of 9.2 g/dL (microcytic hypochromic anemia), and increased platelets of 626 x 10^9^/L. Her CRP (C-reactive protein) was significantly elevated at 319 mg/L (normal range [NR] <10 mg/L) and procalcitonin was 3.9 ng/mL (NR <0.05 ng/mL). Initial treatment included intravenous ceftriaxone, azithromycin, and one fluid bolus of normal saline (NS) 20 mL/kg due to borderline perfusion. Additionally, the polymerase chain reaction (PCR) of the nasal swab that is routinely done for children with respiratory symptoms came back positive for RSV and COVID-19. 

**Figure 2:**
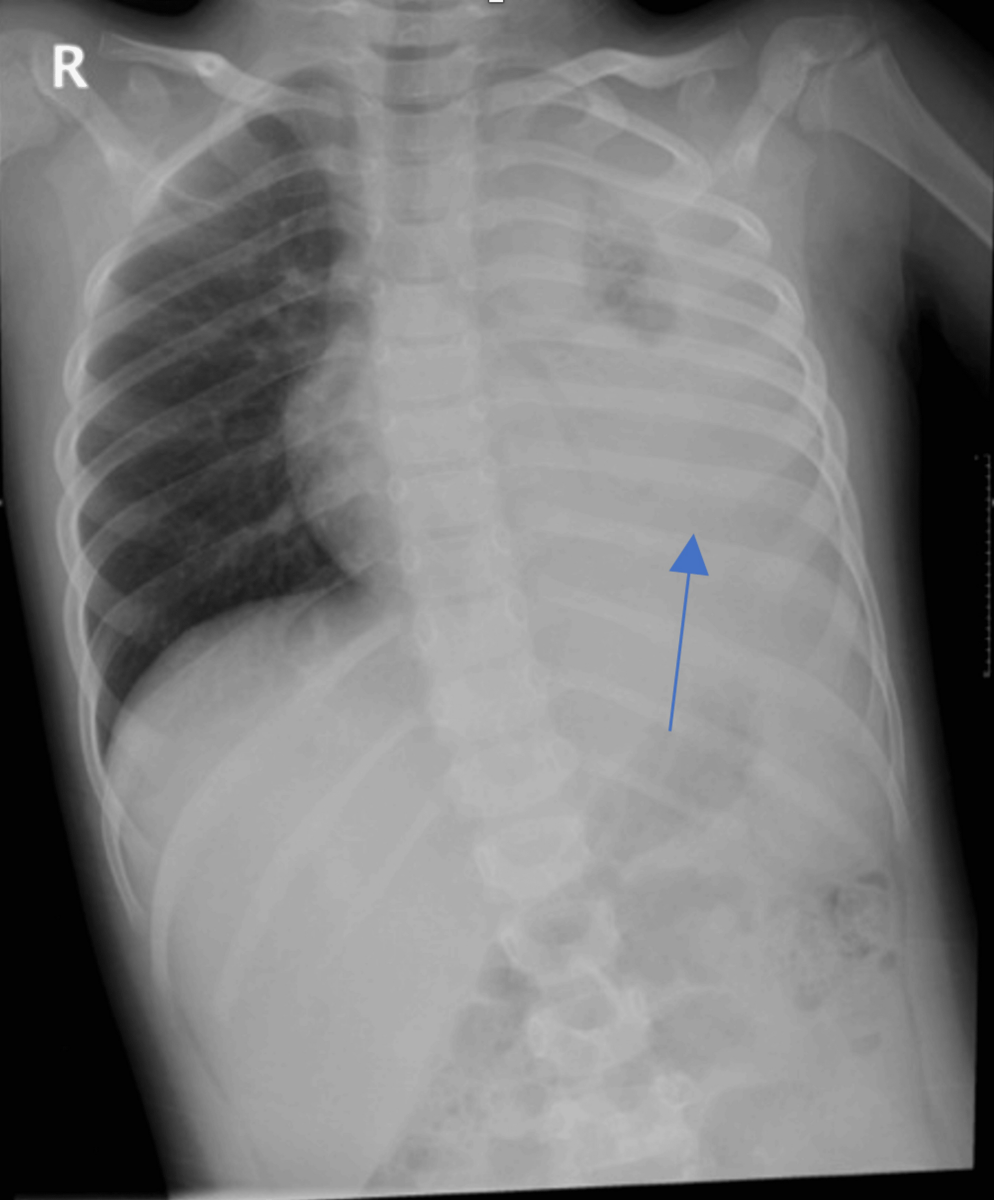
Chest X-ray on PICU Day 1 demonstrating worsening hemogenic opacity in the left side (blue arrow) PICU, Pediatric Intensive Care Unit.

**Figure 3:**
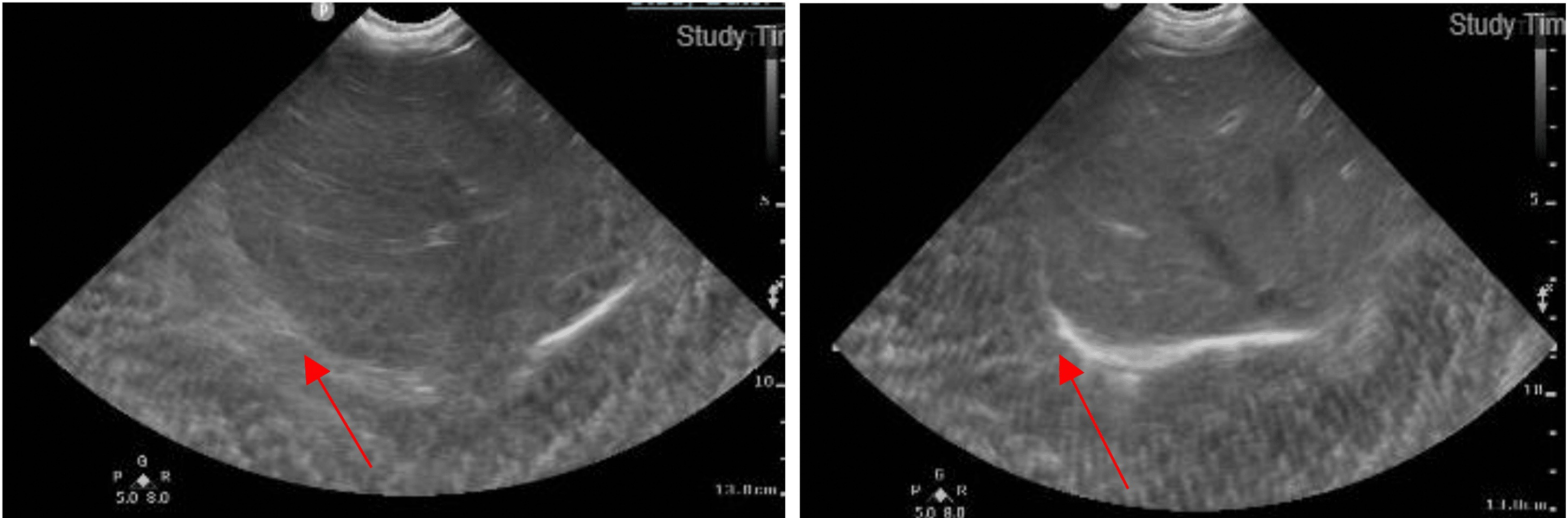
Point-of-care ultrasound (POCUS) showing the upper two-thirds of the left lung with mild effusion with mainly lung disease, and the lower third with moderate pleural effusion with lung disease

The case was diagnosed as complicated pneumonia with para-pneumonic effusion, and she was admitted to the Pediatric Intensive Care Unit (PICU) for further management. Due to the worsening respiratory status and given the PCR results, the treatment regimen was expanded to include intravenous vancomycin and ceftriaxone for five days, pending blood cultures, and dexamethasone (five doses). The repeated chest X-ray (Figure 2) and point-of-care ultrasound (POCUS) (Figure 3) conducted in the PICU confirmed moderate left pleural effusion, mostly involving the lower third of her left lung, but notably without significant septations. 

Consultations were sought from both infectious disease specialists and pulmonologists to ensure a comprehensive approach to the patient's care. The multidisciplinary team agreed on a treatment plan that involved maintaining the current medication regimen, which included antibiotics and supportive therapies. They also discussed the potential need for drainage of the pleural effusion if her condition did not improve or if imaging showed progression. In addition to these measures, the team emphasized the importance of closely monitoring her inflammatory markers, such as CRP and procalcitonin, to evaluate the effectiveness of the treatment and make timely adjustments as needed. 

PICU course 

Our patient remained conscious, alert, and fully oriented with a Glasgow Coma Scale (GCS) score of 15/15. Significant improvement in respiratory status was observed over the course of the PICU stay, with tailored respiratory treatment that included salbutamol, hypertonic saline nebulization, and continuing dexamethasone IV for five days plus two days of methylprednisolone. She was kept on a face mask (6 L per minute) for two days then weaned to room air. A hemoglobin level of 9.4 g/dL was noted, indicating hypochromic microcytic anemia, but as the patient did not manifest hemodynamic issues, no packed red blood cell transfusion was needed. Throughout the stay, the patient remained afebrile and continued a course of vancomycin and ceftriaxone as per the pediatric ID expert opinion. Both blood cultures were negative, and inflammatory markers showed a good response to treatment, with CRP decreasing from 319.130 mg/L to 188.900 mg/L, and procalcitonin dropping from 3.95 ng/mL to 2.66 ng/mL. The treating team assessment concluded that the ongoing RSV/COVID infection was complicated by a bacterial infection and para-pneumonic effusion. A care plan was established to continue antibiotic therapy, with regular monitoring in place for ongoing evaluation. 

General ward course 

**Figure 4:**
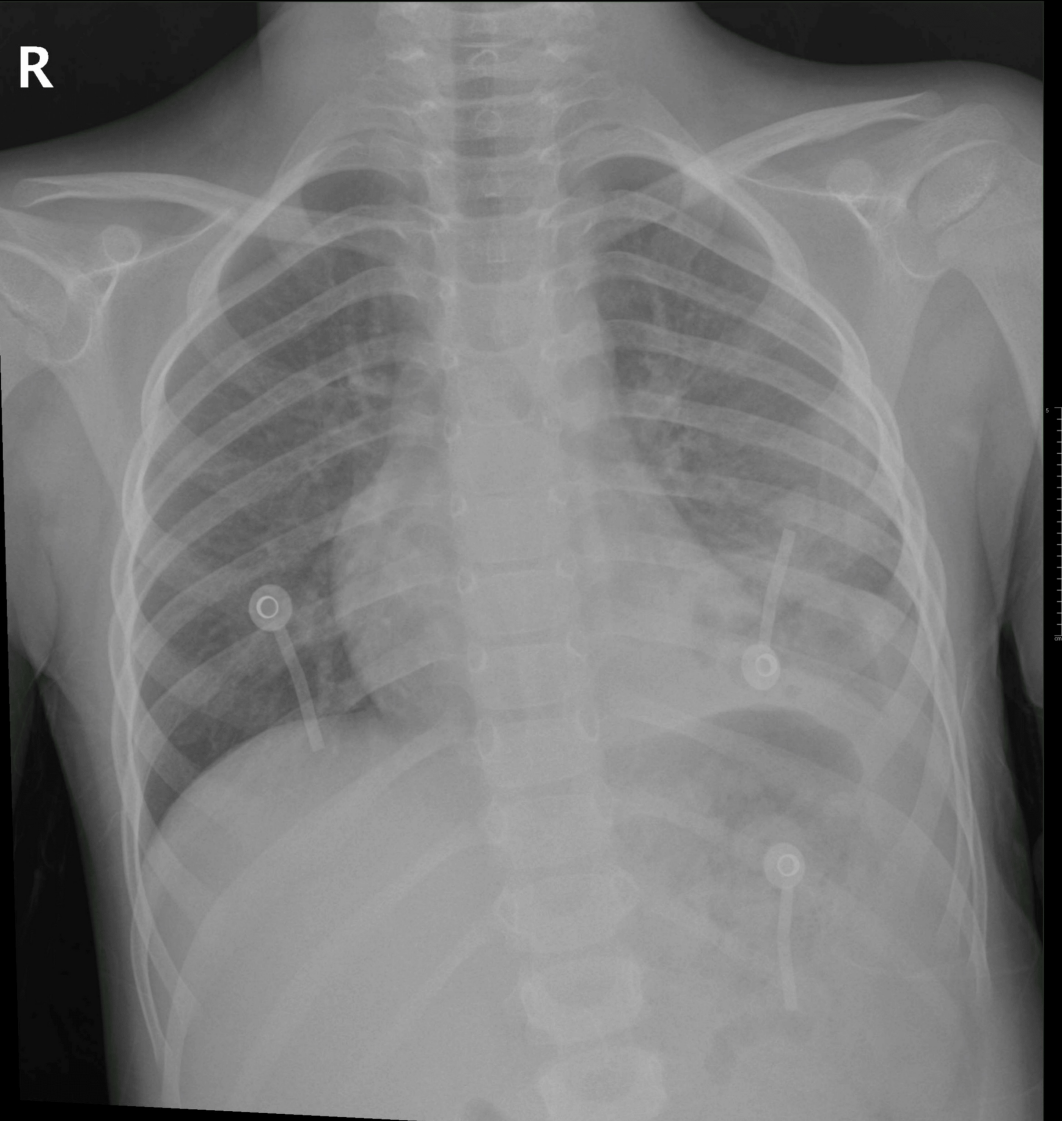
Chest X-ray showing improved aeration of the lung fields

After six days in the PICU, the patient demonstrated significant clinical improvement. She remained afebrile, became more active, and engaged well with the caregivers. Respiratory status continued to stabilize, as evidenced by the follow-up imaging showing a reduction in pleural effusion (Figure 4) with resolved respiratory distress. Decreasing inflammatory markers, with CRP now at 188.900 mg/L and procalcitonin at 2.66 ng/mL, along with negative blood cultures, indicate a positive response to the antibiotic regimen. She completed the parenteral antibiotic course for five days and continued on oral antibiotics (cefuroxime/clindamycin) for two additional days. 

She was discharged home after 29 days in good condition. Follow-up in the outpatient clinic was advised. 

## Discussion

Our case involved dual viral infections (RSV and COVID-19) with a complication of para-pneumonic effusion. The case discussed introduces varicella as a co-existing viral factor alongside COVID-19, emphasizing the potential for different viral interactions that could lead to severe pulmonary pathology. Furthermore, the role of RSV in predisposing patients to bacterial superinfection needs further exploration. Emphasizing mechanisms such as epithelial damage and immune dysregulation caused by RSV can aid in understanding patient vulnerability and guiding appropriate clinical management. Expanding on these points would enhance clarity in diagnosing superinfections in patients with co-infections [6]. Several strategies could mitigate the burden of severe respiratory viral infections among children in the post-COVID-19 era, including rapid bedside detection tools [7]. Rapid detection by the Point-of-Care Multiplex of Respiratory Viruses could be utilized to perform fast (less than 30 min) testing without the use of RNA extraction kits [8]. Early detection of dual respiratory viruses could guide prompt antiviral regimens and suitable isolation measures for these critically ill children. The rarity of the combined varicella and COVID-19 cases underscores the importance of vigilant clinical observation in pediatric respiratory infections with potential co-infections [9]. The rise in other viral respiratory infections among previously healthy children during the post-pandemic season supports the idea of an "immunity debt” with some loss of herd immunity. In this case, the concurrent presence of RSV and COVID-19 complicated her clinical picture, raising questions about the potential for bacterial superinfection in addition to the viral causes. To aid in this differentiation, inflammatory markers such as procalcitonin and CRP were utilized. RSV is commonly associated with empyema, particularly in children, as viral infections can lead to secondary bacterial pneumonia, resulting in empyema in up to 15% of RSV-related hospitalizations. In the study reviewed, bilateral lung involvement was observed in 76.06% of RSV cases, which may predispose patients to complications such as empyema [10]. 

Procalcitonin as an Indicator in our case: A procalcitonin level of 3.95 ng/mL was exhibited. While elevated, this level falls into a range where it provides suggestive rather than definitive evidence of bacterial infection. Procalcitonin levels are generally lower in viral infections, which is why they are sometimes used to guide antibiotic therapy decisions and potentially reduce unnecessary use of antibiotics. However, levels between 0.5 and 2 ng/mL can indicate bacterial infection, with levels above 2 ng/mL considered more indicative of bacterial involvement. Despite the level not being extremely elevated, clinical factors still supported empirical antibiotic therapy while monitoring her response [11]. 

CRP and its role in COVID-19

CRP is another inflammatory marker elevated in both bacterial and viral infections, including COVID-19. In viral infections such as COVID-19, CRP levels can be significantly elevated due to the body's inflammatory response. Studies have shown that high CRP levels in COVID-19 cases can correlate with disease severity, reflecting the level of systemic inflammation rather than indicating a bacterial cause. In our situation, elevated CRP levels mirrored the significant inflammation from her viral infections, specifically COVID-19, and helped assess her overall inflammatory burden [12,13]. Elevated levels of CRP are commonly associated with viral infections, with research showing that about 62.8% of individuals with respiratory viral infections exhibit abnormal CRP levels. Increases in CRP are linked to extended hospital stays and a greater risk of requiring intensive care or mechanical ventilation. Nevertheless, it is important to acknowledge the limitations of CRP as a diagnostic tool. There is often a significant overlap in CRP levels between viral and bacterial infections, which can make accurate diagnosis challenging. Furthermore, CRP responses can vary in different populations, especially in pediatric patients. This variability underscores the necessity of interpreting CRP results carefully, considering the broader clinical context and other diagnostic information for effective patient care [14]. 

A prolonged hospital stay is linked to considerable occupancy of hospital beds, with potential increased complications, and the use of resources that could otherwise be reserved for other healthcare services [15]. The home healthcare-based IV antibiotic program proved to be a safe and effective alternative to in-patient care for managing patients with non-life-threatening infections, with a very low complication rate [16]. Implementing Outpatient Parenteral Antimicrobial Therapy (OPAT) Programs with disposable elastomeric pumps is a new potential solution [17]. Future research should explore the factors contributing to extended pediatric lengths of stay and evaluate the effectiveness of various policies to offer home parenteral antibiotics and enhance resource allocation and management [18]. 

## Conclusions

In the management of this case, the decision to use antibiotics was not solely based on procalcitonin levels but rather on a comprehensive assessment including clinical signs, symptoms, and radiological findings. Procalcitonin levels provided useful information but were one part of broader clinical judgment in managing her condition. The effective reduction in both CRP and procalcitonin levels over time signaled a positive clinical response to her treatment. This case underscores the importance of integrating biomarker data, such as CRP's role in indicating COVID-19 severity, with clinical context to drive appropriate treatment decisions for complex infections. 

The concluding questions pose important considerations regarding the presence of bacterial superinfection alongside a viral infection versus a purely viral infection. To aid clinicians in systematically assessing mixed viral-bacterial infections, it would be beneficial to develop a framework that includes key clinical indicators. These indicators might encompass the patient's clinical history, the nature and duration of symptoms, laboratory results (including inflammatory markers like CRP and procalcitonin), and radiological findings. Integrating this comprehensive assessment can assist in distinguishing between viral infections, bacterial superinfections, or mixed cases, ultimately guiding appropriate treatment strategies.
